# Supplementary material for: Rapid Identity and Quantity CQA Test for Multivalent mRNA Drug Product Formulations
Source: Vaccines (Basel). 2022 Oct 12;10(10):1704. doi: 10.3390/vaccines10101704 (PMC9612012; doi:10.3390/vaccines10101704)
Supplement: Supplementary file 1 [file vaccines-10-01704-s001.zip › vaccines-1936146-Supplementary Materials.pdf]

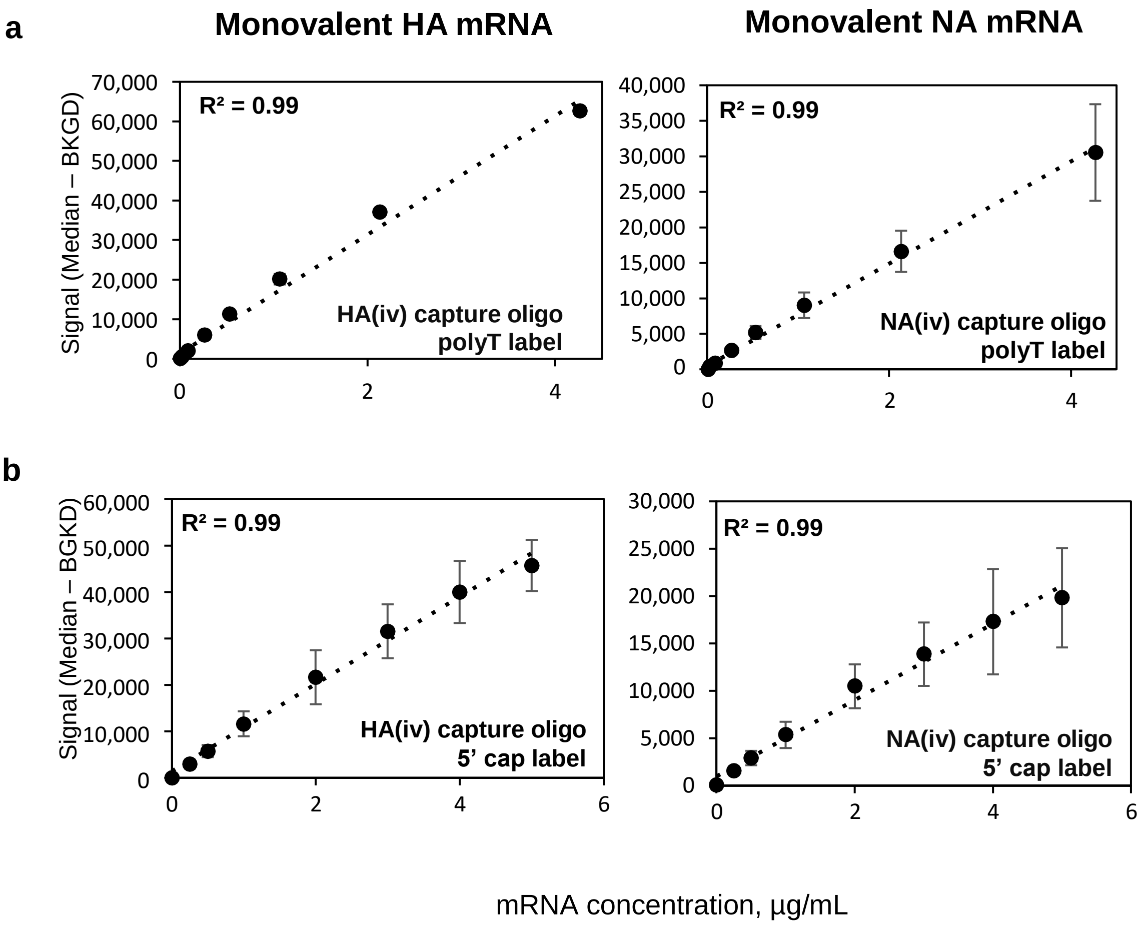

Figure S1. Linearity of response for a variety of capture and detection schemes in monovalent mRNA samples, with HA in left column and NA in right column. a) Coding region capture with polyT label as shown in Figure 1e(i), (b) Coding region capture and labeling with 5' cap antibody label as shown in Figure 1e(ii). Data point are the average of three replicates and error bars indicate ± 1 standard deviation (n=3). R^2 in upper left of each plot is based on a single linear regression.

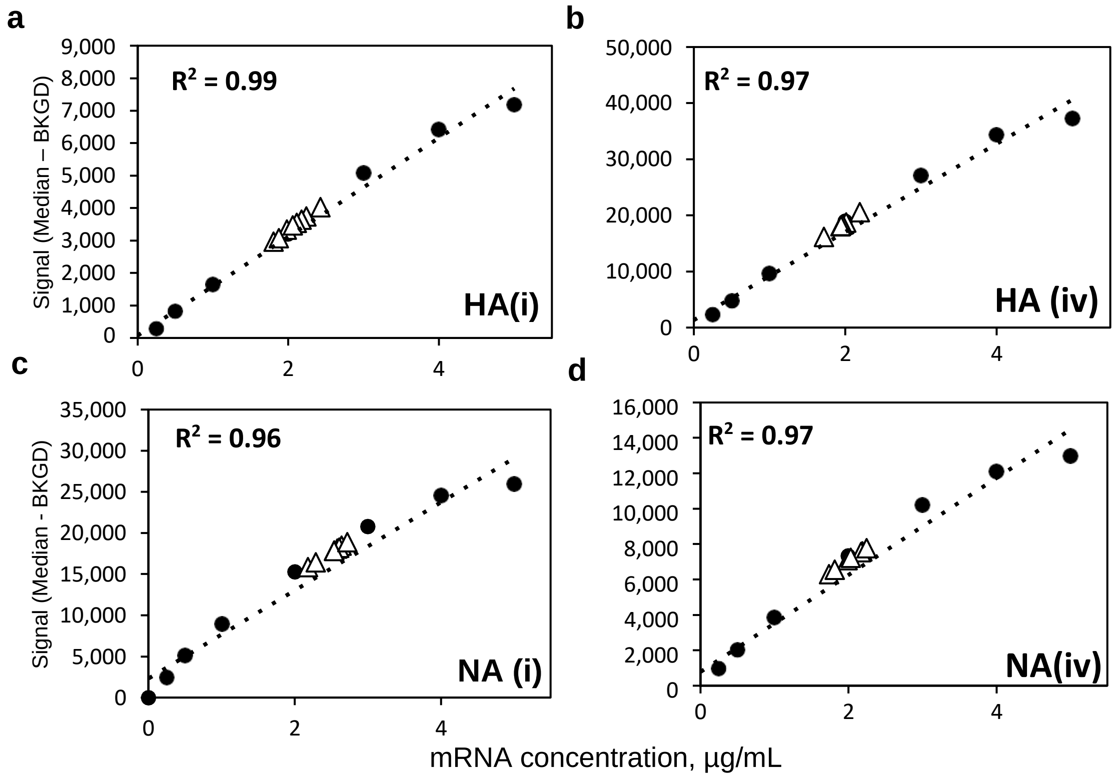

Figure S2. Signal Response curves for monovalent LNP-mRNA samples, labeled with polyT label. 8-pt response curves generated comparing a monovalent HA standard curve (●) to eight replicates of lysed LNP-mRNA (Δ) using (a) HA(i) coding region capture oligo, (b) HA(iv) coding region capture oligo, and for NA mRNA using (c) NA(i) coding region capture oligo, and (d) NA(iv) coding region capture oligo.

Table S1. HA and NA construct sequences

mRNA	Sequence
**HA**	ATGAAGGTGAAGCTGCTGGTGCTGCTGTGCACCTTCACCGCCACCTACGCCGACACCATCTGCATCGG<br>CTACCACGCCAACAACTCCACCGACACCGTGGACACCGTGCTGGAGAAGAACGTGACCGTGACCCACT<br>CCGTGAACCTGCTGGAGAACGGCGGCGGCGGCAAGTACGTGTGCTCCGCCAAGCTGCGCATGGTGAC<br>CGGCCTGCGCAACAAGCCCTCCAAGCAGTCCCAGGGCCTGTTCGGCGCCATCGCCGGCTTCACCGAG<br>GGCGGCTGGACCGGCATGGTGGACGGCTGGTACGGCTACCACCACCAGAACGAGCAGGGCTCCGGC<br>TACGCCGCCGACCAGAAGTCCACCCAGAACGCCATCAACGGCATCACCAACAAGGTGAACTCCGTGAT<br>CGAGAAGATGAACACCCAGTACACCGCCATCGGCTGCGAGTACAACAAGTCCGAGCGgTGCATGAAGC<br>AGATCGAGGACAAGATCGAGGAGATCGAGTCCAAGATCTGGTGCTACAACGCCGAGCTGCTGGTGCTG<br>CTGGAGAACGAGCGCACCCTGGACTTCCACGACTCCAACGTGAAGAACCTGTACGAGAAGGTGAAGTC<br>CCAGCTGAAGAACAACGCCAAGGAGATCGGCAACGGCTGCTTCGAGTTCTACCACAAGTGCAACGACG<br>AGTGCATGGAGTCCGTGAAGAACGGCACCTACGACTACCCCAAGTACTCCGAGGAGTCCAAGCTGAAC<br>CGCGAGAAGATCGACGGCGTGAAGCTGGAGTCCATGGGCGTGTACCAGATCGAGGGCCGC
**NA**	ATGAACCCCAACCAGAAGATCATCACCATCGGCTCCATCTGCATGACCATCGGCATGGCCAACCTGATC<br>CTGCAGATCGGCAACATCATCTCCATCTGGGTGTCCCACTCCATCCAGATCGGCAACCAGTCCCAGATC<br>GAGACCTGCAACCAGTCCGTGATCACCTACGAGAACAACACCTGGGTGAACCAGACCTACGTGAACAT<br>CTCCAACACCAACTTCGCCGCCGGCCAGTCCGTGGTGTCCGTGAAGCTGGCCGGCAACTCCTCCCTGT<br>GCCCCGTGTCCGGCTGGGCCATCTACTCCAAGGACAACTCCGTGCGCATCGGCTCCAAGGGCGACGT<br>GTTCGTGATCCGCGAGCCCTTCATCTCCTGCTCCCCCCTGGAGTGCCGCACCTTCTTCCTGACCCAGG<br>GCGCCCTGCTGAACGACAAGCACTCCAACGGCACCATCAAGGACCGCTCCCCCTACCGCACCCTGATG<br>TCCTGCCCCATCGGCGAGGTGCCCTCCCCCTACAACTCCCGCTTCGAGTCCGTGGCCTGGTCCGCCTC<br>CGCCTGCCACGACGGCATCAACTGGCTGACCATCGGCATCTCCGGCCCCGACTCCGGCGCCGTGGCC<br>GTGCTGAAGTACAACGGCATCATCACCGACACCATCAAGTCCTGGCGCAACAACATCCTGCGCACCCA<br>GGAGTCCGAGTGCGCCTGCGTGAACGGCTCCTGCTTCACCATCATGACCGACGGCCCCTCCGACGGC<br>CAGGCCTCCTACAAGATCTTCCGCATCGAGAAGGGCAAGATCATCAAGTCCGTGGAGATGAAGGCCCC<br>CAACTACCACTACGAGGAGTGCTCCTGCTACCCCGACTCCTCCGAGATCACCTGCGTGTGCCGCGACA<br>ACTGGCACGGCTCCAACCGCCCCTGGGTGTCCTTCAACCAGAACCTGGAGTACCAGATGGGCTACATC<br>TGCTCCGGCGTGTTCGGCGACAACCCCCGCCCCAACGACAAGACCGGCTCCTGCGGCCCCGTGTCCT<br>CCAACGGCGCCAACGGCGTGAAGGGCTTCTCCTTCAAGTACGGCAACGGCGTGTGGATCGGCCGCAC<br>CAAGTCCATCTCCTCCCGCAAGGGCTTCGAGATGATCTGGGACCCCAACGGCTGGACCGGCACCGACA<br>ACAAGTTCTCCATCAAGCAGGACATCGTGGGCATCAACGAGTGGTCCGGCTACTCCGGCTCCTTCGTG<br>CAGCACCCCGAGCTGACCGGCCTGGACTGCATCCGCCCCTGCTTCTGGGTGGAGCTGATCCGCGGCC<br>GCCCCGAGGAGAACACCATCTGGACCTCCGGCTCCTCCATCTCCTTCTGCGGCGTGAACTCCGACACC<br>GTGGGCTGGTCCTGGCCCGACGGCGCCGAGCTGCCCTTCACCATCGACAAG

Table S2. Oligonucleotide sequences designed

Type	Name	Sequence (5’ → 3’)
Capture Oligo	**HA(i)**	TGTTGGCGTGGTAGCCGATGC
	**HA(ii)**	CCGCTCGGACTTGTTGTACTC
	**HA(iii)**	TGCACTCGTCGTTGCACTTGTG
	**HA(iv)**	CGTCGATCTTCTCGCGGTTC
	**NA(i)**	GATGTTGCCGATCTGCAGGATC
	**NA(ii)**	GTTGTTGCGCCAGGACTTGAT
	**NA(iii)**	GTTCTGGTTGAAGGACACCCAGG
	**NA(iv)**	TGATGGAGAACTTGTTGTCG
	**polyT**	TTTTTTTTTTTTTTTTTTTTTTTTTTTTTT
Label	**polyT**	TTTTTTTTTTTTTTTTTTTTTTTTTTTTTT
	**HA nt741-760**	CGTCGATCTTCTCGCGGTTC
	**NA nt1149-1168**	TGATGGAGAACTTGTTGTCG
